# The Involvement of Laminin in Anti-Myocardial Cell Autoimmune Response in Murine Chagas Disease

**DOI:** 10.1155/2000/17424

**Published:** 2000

**Authors:** Suse Dayse Silva-Barbosa, Wilson Savino

**Affiliations:** 1 Laboratory on Thymus Research Department of Immunology Institute Oswaldo Cruz Foundation Oswaldo Cruz Rio de Janeiro Brazil; 2 Laboratory on Cell Markers Center for Bone Marrow Transplantation National Cancer Institute Rio de Janeiro Brazil; 3 Laboratory on Thymus Research Department of Immunology Oswaldo Cruz Institute, Oswaldo Cruz Foundation Ave. Brasilo 4365 Manguinhos Rio de Janeiro 21045-000 Brazil

## Abstract

The pathogenesis of chronic chagasic cardiomyophathy associated with Chagas disease is still controversial, although evidence indicates a T cell-dependent autoimmune process. Using a mouse model for chronic Chagas disease, we previously evidenced that hearts grafted within the ears of Trypanosoma cruzi infected syngeneic recipients were rejected through a CD4^+^ T cell-dependent mechanism. Moreover, we showed that such a process was dependent on laminin-mediated interactions, since it could be abrogated by anti-laminin or anti-laminin receptor antibodies. In this review the same passive cell transfer model is considered for discussion: the participation of the laminin alteration in the composition of the inflammatory infiltrate formed in response to the antimyocardial autoreactive CD4^+^ T cells, as well as the presence of laminin-binding cytokines. Finally we suggest the existence of a relationship between the inflammatory infiltrate, the laminin contents and deposition of pro-inflammatory laminin-binding cytokines, which may act in concert during the generation of Chagas disease- related cardiomyophathy.


*Developmental Immunology*, 2000, Vol. 7(2-4), pp. 293-301
Reprints available directly from the publisher
Photocopying permitted by license only

© 2000 OPA (Overseas Publishers Association)
N.V. Published by license under the
Harwood Academic Publishers imprint,
part of the Gordon and Breach Publishing Group.
Printed in Malaysia

### The Involvement of Laminin in Anti-Myocardial Cell Autoimmune Response in Murine Chagas Disease

SUSE DAYSE SILVA-BARBOSA[ab*] and WILSON SAVINO[a]

[a]*Laboratory on Thymus Research, Department of Immunology and Institute Oswaldo Cruz, Foundation Oswaldo Cruz, Rio de Janeiro – Brazil and* [b]*Laboratory on Cell Markers, Center for Bone Marrow Transplantation, National Cancer Institute, Rio de Janeiro – Brazil*

The pathogenesis of chronic chagasic cardiomyophathy associated with Chagas disease is still controversial, although evidence indicates a T cell-dependent autoimmune process. Using a mouse model for chronic Chagas disease, we previously evidenced that hearts grafted within the ears of Trypanosoma cruzi infected syngeneic recipients were rejected through a $CD4^+$ T cell-dependent mechanism. Moreover, we showed that such a process was dependent on laminin-mediated interactions, since it could be abrogated by anti-laminin or anti-laminin receptor antibodies. In this review the same passive cell transfer model is considered for discussion: the participation of the laminin alteration in the composition of the inflammatory infiltrate formed in response to the antimyocardial autoreactive $CD4^+$ T cells, as well as the presence of laminin-binding cytokines. Finally we suggest the existence of a relationship between the inflammatory infiltrate, the laminin contents and deposition of pro-inflammatory laminin-binding cytokines, which may act in concert during the generation of Chagas disease-related cardiomyophathy.

Increasing evidence demonstrates that a large variety of cellular activities depend on extracellular matrix (ECM) components, a network of proteins and glycosaminoglycans filling the intercellular spaces of tissues. This specialized structure is involved not only in the support and maintenance of tissue architecture but also as signaling molecules that interact with cellular receptors to convey outside-in signals (Myamoto and Yamada, 1995). Particularly regarding the immune response, recognition of ECM moieties by lymphocytes is a critical event that regulates the traffic and also influences antigen recognition. In this respect a particular ECM component, namely laminin appears to be one the most potent cell adhesion molecules. Laminin is a family of heterotrimeric glycoproteins, whose prototypical form is composed by three chains ($\alpha$1,$\beta$1,$\gamma$1) which form a cross- shaped molecule with three short arms and one long arm (Burgeson et al., 1994). At least eight laminin chains coded by distinct genes have been identified and can form different heterotrimeric isoforms with specific chain assembly and localization, which appears to be correlated to cell and tissue type (for review see Timpl and Brow, 1994).

As the major structural element of all basement membranes, including those of cardiac and skeletal muscles cells (Ehrig et al., 1990), various biological activities have been attributed to laminin. Cell adhesion is one important biological response to this ECM glycoprotein, being mediated by specific cell surface

* Present address and correspondence: Suse Dayse Silva Barbosa, Laboratory on Thymus Research, Department of Immunology, Oswaldo Cruz Institute, Oswaldo Cruz Foundation, Ave. Brasilo 4365 – Manguinhos, 21045–000 -, Rio de Janeiro – Brazil. Tel: (55) (21) 506–6707 – Fax: (55) (21) 509–2121 – e-mail: suse@gene.dbbm.fiocruz.br receptors; several of them belonging to the integrin family. These integrins, identified in a variety of cells, are transmembrane protein complexes consisting of noncovalently associated α and β subunits. Currently sixteen α and eight β integrin subunits, encoded by different genes, have been described to form distinct heterodimers. Among these, most laminin-responsive cells utilize β1 integrin in association with α1, α2, α3, α6 or α7 subunit (Kramer et al., 1993; Dewel et al., 1994; Brown et al., 1994; Chung-Chen et al., 1996). Some of these receptors can recognize others ECM components although the α6β1 integrin (also named VLA-6) is proposed to be a laminin specific receptor.

The direct effect of laminin upon *in vivo* effector immune response was previously established by experiments using a rat model of heart transplantation. Rejection of allogeneic heart grafts in rats could be abrogated by injection of anti-laminin antibodies, indicating that such an immune intervention can affect the migratory pattern of passenger leukocytes (de Sousa *et al*, 1991).

In this review, we shall discuss the role of laminin, in an immune reaction against myocardial tissue, induced by autoreactive cells from *Trypanosoma cruzi* chronically infected mice. Yet, before discussing the issue related to the involvement of laminin in chronic chagasic cardiomyophathy, it is worthwhile to provide a short general background on the immunopathology of Chagas disease.

### CHRONIC CHAGAS CARDIOMYOPATHY: AN EXAMPLE OF T CELL DEPENDENT AUTOIMMUNE DISEASE

Chagas disease, caused by the flagellate protozoan *Trypanosoma cruzi*, is an endemic parasitic disease affecting 18–20 million individuals in Latin America. In the chronic phase of the disease, the presence of cardiac disorders with consequent heart failure, relates to death in about 20% of the patients (Brener and Gazzinelli, 1997).

Cardiomyopathy associated with chronic Chagas disease is characterized by inflammation and degeneration of cardiac muscle as a consequence of the infection. Nevertheless, the pathogenesis of this cardiomyopathy is still controversial. In the past, the apparent hardness to find *T. cruzi* in heart chronic inflammatory lesions argued against a direct participation of the parasite in the tissue damage. Nowadays, with introduction more sensitive techniques, *T. cruzi* DNA and parasite-derived antigens have been reported within the hearts of chronically affected individuals (Jones et al., 1993; Higushi et al., 1993; Lane et al., 1997). This observation reinforces the significance of the parasite in the development of chronic Chagas myocarditis, although additional mechanisms could be involved in the heart lesion process.

We previously designed a number of experiments to determine in *T. cruzi* infected mice, which lymphocyte subsets were involved in experimental cardiomyopathy related to the chronic phase of the disease. Using a previous established model of heart transplantation into the subcutaneous tissue of the ear, we demonstrated that syngeneic heart tissues from newborn donors (which are normally accepted in syngeneic conditions) were usually rejected when grafted within the ears of *T. cruzi* chronically infected syngeneic recipients. In this system, the treatment of infected animals with anti-CD4, but not with anti-CD8 monoclonal antibody abrogated this rejection, thus unrevealing the crucial role of $CD4^+$ T cells in the process. Moreover, when purified $CD4^+$ T cells from chagasic animals were transferred to the ears of naive normal syngeneic recipients, the transplanted hearts were rejected four days after injection, with completely absorption in seven days post cell transfer (Ribeiro dos Santos et al., 1992). Accordingly, the injection of $CD4^+$ cells from chronically infected mice is followed by a extensive myocyte necrosis of the grafted tissue, associated to a severe and diffuse mononuclear cell infiltrate.

Conjointly, these *in vivo* data suggest that autoimmune mechanisms are involved in the pathogenetic process of heart tissue damage. In fact, recent studies conducted in human Chagas disease indicate a T cell-dependent autoimmune process, which may be related to the generation of the myocarditis associated with the chronic phase. Such autoreactivity appears to be derived, at least in part, from molecular mimicry, since the presence of a specific fragment of the cardiac (but not skeletal) myosin can induce proliferation of $CD4^+$ T cell clones derived from a chagasic patient (Cunha-Neto et al., 1996).

In this context an extensive tissue damage induced by the massive presence of parasite into the heart during acute phase of Chagas disease, together with its persistence in the chronic phase, favor the hypothesis that autoreactive clones against products from myocardial tissue could be maintained. Then, disturbances leading to the break of self-tolerance and modifying the microenvironment can be involved in the development of this parasite-induced autoimmune process.

### POSSIBLE INVOLVEMENT OF LAMININ IN THE AUTOIMMUNE RESPONSE FOUND IN CHAGAS DISEASE

### Changes of laminin contents in the context of T. cruzi infection: lessons from transplanted animals

The analysis of heart biopsies from patients in different clinical stages of the Chagas disease, cardiac with or without heart failure, demonstrated that inflammation is more severe in groups presenting heart failure. In the chronic cardiomyopathy, a foccused or diffuse mononuclear cell infiltrate is observed, positively correlating with regions of myonecrosis, interstitial fibrosis and rare fibers with intracellular parasite (see review Brenner and Gazinelli, 1997).

Evidence for the contribution of laminin to heart inflammatory response in Chagas disease was firstly provided by the detection an increase in laminin deposits intermingled with myocardial fibers in heart tissue from experimental models (Andrade et al., 1989; Miley et al., 1993; Sanchez et al., 1993).

In a second vein, a series of studies indicates that laminin and VLA-6 expression in lymphoid organs can be altered along with infectious diseases. In peripheral T cells from chagasic animals, we evidenced an increase in VLA-6 expression, not only in the acute, but also in the chronic phase of the *T. cruzi* infection (Lima-Quaresma et al., *in preparation*). For example, in chronically infected mice, we found a rise in both absolute and relative numbers of $CD4^+$ T lymphocytes bearing high density of VLA-6 (Silva-Barbosa et al., 1997). These findings, together with the data showing an increase in laminin expression within autologous heart tissue (Andrade et al., 1989), indicate that VLA-6/laminin interactions may be relevant for the developing of the carditis that takes place in chagasic mice and humans.

Interestingly, such a VLA-6/laminin interaction appears to be a broader event, also occuring in typical allogeneic T cell dependent immune responses. In fact, rejection of allogeneic heart grafts in rats could also be blocked by *in vivo* treatment with anti-laminin antibodies (Kupiec-Wieglinski and de Sousa, 1991), a result that we have also seen in mice that received allogeneic heart grafts in the ear lobes (Riederer et al, manuscript in preparation).

In experimental *T. cruzi* infection, once we established that heart grafts were rejected through a $CD4^+$ T cell-dependent autoreactive mechanism, we searched whether or not such a process was dependent on laminin-mediated interactions. Consistently with our previous data, we showed that in the transplant heart model, transfer of $CD4^+$ T cells from infected animals elicited an abnormally dense laminin network in the areas surrounding the myocardial cells and externally to the grafts accompanies the rejection process. Moreover, we found pre-labeled injected cells within the grafts, in sites rich in laminin (Silva-Barbosa et al., 1997). Conjointly, these results prompted us to investigate a putative involvement of laminin and VLA-6 upon the influx of effector cells into the lesion. To approach this issue we treated the recipients' ears with an single dose of anti-laminin monoclonal antibody (mAb) adjacent to the graft, and prior to passive transfer of spleen-derived $CD4^+$ T cells from chagasic mice. This treatment significantly blocked heart rejection. Importantly, incubation of $CD4^+$ T cells from infected animals with the anti-laminin receptor mAb before cell transfer, also resulted in abrogation of graft rejection, as ascertained by direct monitoring of heart beats and of macroscopic aspect

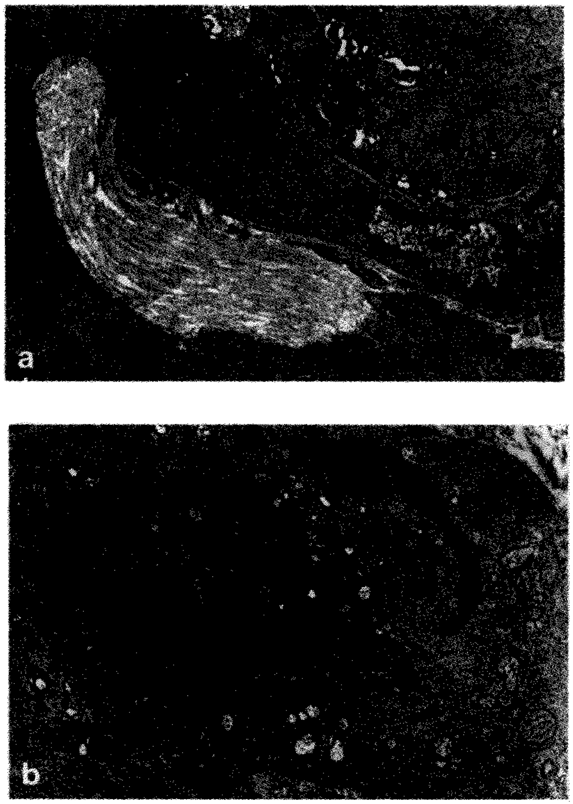

FIGURE 1 Ultrastructural patterns of newborn transplanted hearts after injection of spleen-derived CD4$^+$ T cells from infected mice previously treated with with the anti-laminin receptor mAb (*a*) or unrelated rat immunoglobulins (*b*). When rejection is abrogated by the anti-VLA-6 mAb, normal elongated myocardial cells with myofibrils arranged in parallel bundles are seen (panel *a*). Such an arrangement is completely lost in rejected hearts, as seen in panel *b*. x14,000

of the graft, as well as histological and ultrastructural evaluation (Fig. 1).

In both blocking protocols, clusters of donor cells were seen outside (but not within) the grafted tissue (Fig. 2). In the non-rejected tissue, myocardial cells were well preserved and the intragraft inflammatory infiltrate was very discrete. These findings evidence that a laminin/VLA-6 interaction may be involved in triggering the antimyocardial autoreactive process by driving the influx of CD4$^+$ T cells to the heart.

It should be noted that, as compared to syngeneic grafts that received normal spleen-derived CD4$^+$ T cells, hearts undergoing rejection showed a denser deposition of laminin in the areas surrounding the myocardial cells. Such a denser laminin-containing network was also detected externally to the graft, including the connective tissue between the cartilage and epidermis. The animals receiving the anti-laminin mAb prior to transfer of CD4$^+$ T cells from chronically infected mice, exhibited a laminin-bearing network which was markedly decreased, not only in areas adjacent to myocardial cells but also externally to the graft.

We then studied the relationship between the laminin contents and the inflammatory process. For that we evaluated the ear lobes of transplanted mice, treated with either the anti-laminin or the unrelated antibody mAb (before injection of CD4$^+$ T cells from *T. cruzi* chronically infected mice). The same progressive laminin deposition was detected when we analyzed the cellular infiltrate within the grafts. The heart transplant area from animals pre-treated with unrelated antibody consisted of CD4$^+$ T cells, macrophages, as well as scarce dendritic and CD8$^+$ T cells. This pattern contrasted with the findings seen in anti-laminin mAb treated mice, in which the numbers of lymphocytes, macrophages and dendritic cells were always smaller.

### Cytokine-laminin interactions: clues to understand the pathophysiology of chagasic cardiopathy?

It is interesting to note that various endogenous stimuli can modulate the expression of laminin and VLA-6, with consequences on the lymphocyte-microenvironmental cell interactions. Such stimuli comprise growth factor and cytokines, as for example interferon-γ that modulates ECM production and ECM receptor expression by thymic epithelial cells, with consequences on the degree of thymocyte adhesion (Lagrota-Cândido et al., 1996).

In a second vein, increasing evidence suggests that laminin as other ECM molecules play a further role in the functioning of the immune system through their ability to bind some cytokines and consequently increase their concentration of these molecules at a

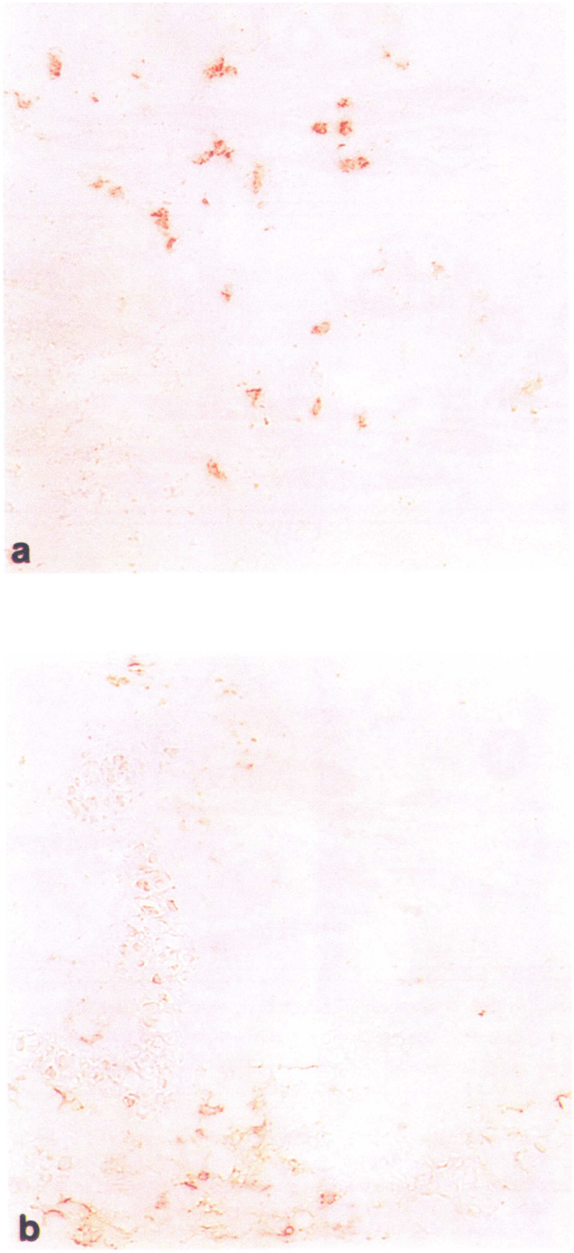

FIGURE 2 Immunohistochemical localization of CD4$^+$ cells in sections of neonatal transplanted hearts areas. Panel *a* represents the CD4$^+$ expression in a graft that received cells treated with unrelated rat immunoglobulins. A scattered mononuclear infiltrate of these cells can be seen within and surrounding the graft. Such signs of rejection are not seen when graft area is treated with the anti-laminin antibody before cell transfer (panel *b*). In contrast, in this case clusters of CD4$^+$ cells are found outside the graft. Magnifications: x250 (see Color Plate XXI at the back of this issue)

given site of immunological activity. Recent literature actually demonstrates that various ECM components are able to bind to cytokines (Yamagushi et al., 1990; Lantz et al., 1991; Tanaka et al., 1993). Presumably, the binding of cytokines and growth factors to ECM molecules provides the necessary signal(s) to anchorage of responsive cells. For example, is has been shown that IFN-γ bind to matrigel (a complex structure formed by various ECM components of basement membranes) particularly through the heparan sulfate moiety (Lortat-Jakob et al., 1991). Additionally, the binding of a cytotoxic T-cell line to laminin leads to the enhancement of IFN-γ mRNA expression, and such effect is blocked by anti-VLA6 mAb (Li et al., 1998). Furthermore, TNF-α can bind to laminin, and such an event amplifies the pro-adhesive effect of the cytokine upon lymphocytes (Hershkoviz et al., 1995).

Recent reports strongly support a role of cytokines in the genesis of the autoimmune process and in determining the susceptibility or resistance to infection (Romagnani, 1996; Lafaille et al., 1997; Fresno et al., 1997; Trembleau et al., 1995). Studies performed in experimental models of Chagas disease have demonstrated that *in vivo* treatment with anti-IFN-γ or anti-TNF-α mAb renders the animal more susceptible to acute infection (Silva et al., 1992, 1995). In addition, in chronically infected mice, activated lymphocytes were detected; being associated with the production of both inflammatory and anti-inflammatory cytokines in the chagasic hearts (Zhang & Tarleton, 1996). Moreover, the high production of IFN-γ by peripheral blood mononuclear cells, as well as the detection of IFN-γ and TNF-α in cardiac autopsies from chronically chagasic patients (Reis et al., 1993; Dutra et al., 1997; Bahia-de-Oliveira et al., 1998) may be related to the development of heart damage in chronic chagasic patients. Additionally, studies in heart biopsies showed that IFN-γ was the most abundant cytokine produced by T cells lines obtained from chronic Chagasic patients exhibiting cardiomyopathy (Cunha-Neto et al., 1998).

The mechanisms involved in trapping these cytokines so that to concentrate them in the lesions,

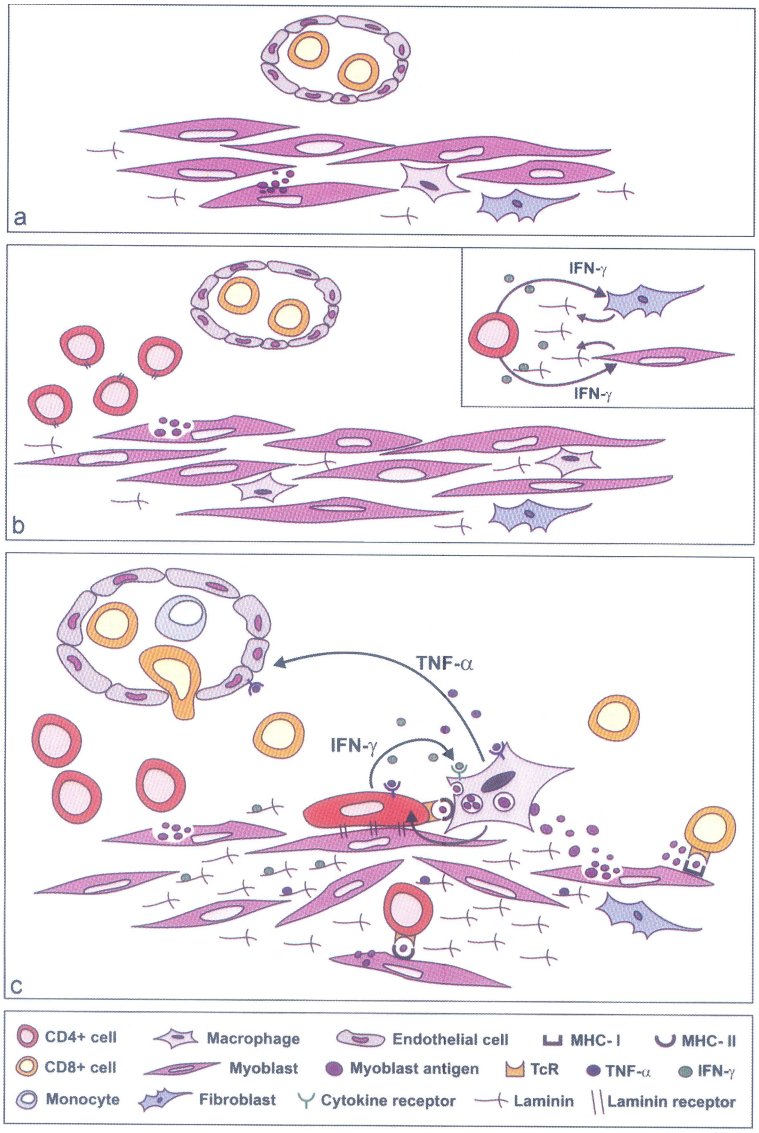

FIGURE 3 Hypothetical scheme on the mechanisms involved in heart graft rejection mediated by $CD4^+$ T cells from chagasic mice. Panel ***a*** depicts a heart transplanted area before injection of splenic $CD4^+$ T cells derived from infected mice, showing the distribution of myoblast cells, resident fibroblasts and macrophages, laminin and myoblast antigens. Injection of $CD4^+$ cells from infected mice (in red) can convey the local production of IFN-γ. Additionally, IFN-γ is possibly up-regulating laminin production by stromal cells and/or cardiomyocytes (panel ***b***). Once laminin content is locally augmented, it favors cell arrival, so that the leukocyte recruitment triggered by IFN-γ should be further enhanced, through the well defined haptotatic effect of this ECM protein. Moreover, IFN-γ can activate macrophages to produce TNF-α and increase their expression of MHC gene products (panel ***c***). In response to the transplantation process and/or injection of $CD4^+$ cells, cardiac antigens can be exposed and presented by local antigen presenting cells. On the other hand, the binding of IFN-γ and TNF-α to laminin, not only increases their local concentrations, but also favor the activation of resident macrophages. This is relevant in the context discussed herein, since the presence of activated TNF-α secreting macrophages, can also act upon endothelial cells, increasing the expression of adhesion molecules and recruiting circulating cells from the recipient animal. Conjointly, these stimuli would amplify and maintain the cardiac inflammatory lesion. Since the *in vivo* treatment with anti-laminin mAb revealed a progressive decrease in cell infiltration associated with a diminished deposition of laminin and laminin-binding inflammatory cytokines, such a treatment would, not only decrease the intra-graft influx of $CD4^+$ cells, but also diminish the secretion/sequestration of IFN-γ and TNF-α, as compared to the pattern observed in rejecting control heart grafts (see Color Plate XXII at the back of this issue)

allowing them to be presented to the various cell types of the immune system, have not been investigated. Laminin is one candidate molecule to play such a role, since it was shown to be able to bind both IFN-γ and TNF-α (Hershkoviz et al., 1993; Li et al., 1998). In this respect, we recently defined the distribution pattern these laminin-binding cytokines in response to the presence of antimyocardial autoreactive $CD4^+$ T cells, in heart tissue previously grafted to syngeneic normal recipients. We further compared these parameters, either in the absence or in the presence of a local anti-laminin mAb treatment. Immunostaining revealed IFN-$\gamma^+$ cells and TNF-$\alpha^+$cells externally to and within the grafts after $CD4^+$ T cell transfer. Additionally, a fibrillary immunostaining for IFN-γ and TNF-α was detected, and by confocal microscopy evaluation we found that it was co-localized with laminin. In contrast, when we analyzed anti-laminin treated animals, the intra-graft labeling for these cytokines was largely diminished along with the days post-transplantation. Overall, the *in vivo* anti-laminin immune intervention resulted in a decrease of laminin contents within and surrounding the graft, that paralleled a decrease in the cellular infiltrate and a local diminution of the laminin-binding cytokines, IFN-γ and TNF-α.

The role of TNF-α was also investigated in allogeneic heart graft in rats: injection of the cytokine enhanced graft rejection whereas treatment with anti-TNF-α antibodies blocked rejection of the transplanted organ (Coito et al., 1994, 1995). However, in this experimental condition laminin deposition and co-localization with TNF-α was not checked during rejection or during its abrogation by anti-TNF-α treatment.

### CONCLUDING REMARKS

Taken together, the data discussed above lead led to the hypothesis that an effective response could be resulting to the balance between soluble factors, proteins of extracellular matrix and the cellular infiltrate formed in response to this "cross-talk". The studies performed with the $CD4^+$ T cell transfer into ears containing syngeneic hart grafts allowed to propose a model to explain the role of laminin as part of the mechanism of graft rejection triggered by transferred cells (Fig. 3).

The concept emerging from the studies discussed so far is that laminin\VLA-6 interaction can be regarded as part of the general control mechanism that governs migration of T lymphocytes, including their interaction with microenvironmental products. Moreover, such notion appears be suitable for normal and pathological states, including autoimmune diseases. In this respect, one can envision such a laminin/VLA-6 interaction as potential target for immuneintervention.

Lastly, since further ECM components do play a role in T cell physiology, it is predictable that interactions involving other ECM ligands and their corresponding receptors (for example fibronectin versus VLA-4 and/or VLA-5) may be involved in the behaviour of activated T cells, independently of the fact that they recognize self or nonself antigens.

### *References*

Andrade S.G., Grimaud J.A., and Stocker-Guerret S. (1989). Sequencial changes of the connective matrix components of the miocardium (fibronectin and laminin) and evolution of cardiac fibrosis in mice infected with *T. cruzi*. Am J Trop Med Hyg 40:252–260.

Bahia-Oliveira L.M., Gomes J.A., Rocha M.O., Moreira M.C., Lemos E.M., Luz Z.M., Pereira M.E., Coffman R.L., Dias J.C., Cançado J.R., Gazzinelli G., and Correa-Oliveira R. (1998). IFN-gamma in human Chagas' disease: protection or pathology? Braz J Med Biol Res 31:127–131.

Brener Z., and Gazzinelli R.T. (1997). Immunological control of *Trypanosoma cruzi* infection and Pathogenesis of Chagas disease. Int Arch Allergy Immunol 114:103–110.

Brown J.C., Wiedemann H., and Timpl R. (1994). Protein binding and cell adhesion properties of two laminin isoforms (AmB1eB2e, AmB1sB2e) from human placenta. J Cell Sci 199107:329–38.

Burgeson R.E., Chiquet M., Deutzmann R., Ekblom P., Engel J., Kleiman H.K., Martin G.R., Ortonne J-P., Paulsson M., Timpl R., Tryggvason K., Yamada Y., and Yurchenco P.D. (1994). A new nomenclature for the laminins. Matrix Biol 14:209–211.

Coito A.J., Binder J., Brown L.F., de Sousa M., van De Water L., and Kuppiec-Weglinski J.W. (1995). Anti-TNF-α treatment down-regulates the expression of fibronectin and decreases cellular infiltration of cardiac allografts in rats. J Immunol 154:2949–2958.

Yao C.C., Ziober B.L., Squillace R.M., and Kramer R.H. (1996). α7 integrin mediates cell adhesion and migration on specific laminin isoforms. J Biol Chem 271: 25598–25603.

Cunha-Neto E., Coelho V., Guilherme L., Fiorelli A., Stolf N., and Kalil J. (1996). Autoimmunity in Chagas' disease. Identification of cardiac myosin-B13 *Trypanosoma cruzi* crossreactive T cell clones in heart lesions of a chronic Chagas' cardiomyopathy patient. J Clin Invest 98:1709–1712.

Cunha-Neto E., Rizzo L.V., Albuquerque F., Abel L., Guilherme L., Bocchi E. Bacal F., Carrara D., Ianni B., Mady C., and Kalil J. (1998). Cytokine production profile of heart-infiltrating T cells in Chagas' disease cardiomyopathy. *Braz J Med Biol Res* 31(1):133–137.

de Sousa M., Tilney N.L., and J. W. Kupiec-Weglinski. (1991). Recognition of self within self: specific lymphocyte positioning and the extracellular matrix. Immunol. Today 12:262–265.

Dewel G.O, de Melker A.A., Hogervorst F., Jaspars L.H., Fles D.L.A., Kuikman I., Lindblon A., Paulsson M., Timpl R., and Sonnemberg A. (1994). Distinct and overlapping specificities of the $\alpha3A\beta1$ and $\alpha6A\beta1$.

Dutra W.O., Gollob K.J., Pinto-Dias J.C., Gazzinelli G., Correa-Oliveira R., Coffman R.L., and Carvalho-Parra J.F. (1997). Cytokine mRNA profile of peripheral blood mononuclear cells isolated from individuals with *Trypanosoma cruzi* chronic infection. Scand J Immunol 45:74–80.

Ehrig K., Leivo I., Argraves W.S., Ruoslahti E., and Engvall E. (1990). Merosin, a tissue-specific basement membrane protein, is a laminin-like protein. Proc Natl Acad Sci U S A 87(9):3264–8.

Fresno M., Kopf M., and Rivas L. (1997). Cytokines and infectious diseases. Immunol. Today 18:56–58.

Hershkoviz R., Gilat D., Miron S., Mekori Y.A., Aderka D., Wallach D., Vlodavsky I., Cohen I.R., and Lider O. (1993). Extracellular matrix induces tumor necrosis factor -$\alpha$ secretion by an interaction between resting rat $CD4^+$ T cells and macrophages. Immunology 78:50–57.

Hershkoviz R., Goldkorn I., and Lider O. (1995). Tumor necrosis factor-$\alpha$ interacts with laminin and functions as a pro-adhesive cytokine. Immunology 85:125–130.

Higuchi M.L., Brito T., Reis M.M., Barbosa A., Bellotti G., Pereira-Barreto A.C., and Pileggi F. (1993). Correlation between *Trypanosoma cruzi* parasitism and myocardial inflammatory infiltrate in human chronic chagasic myocarditis: Light microscopy and immunohistochemical findings. Cardiovasc Pathol 2: 101–106.

Jones E.M., Colley D.G., Tostes S., Lopes E.R., Vnencak-Jones C.L., and McCurley T.L. (1993). Amplification of a Trypanosoma cruzi DNA sequence from inflammatory lesions in human chagasic cardiomyopathy. Am J Trop Med Hyg 48:348–357.

Kramer R.H., Enenstein J., and Walet N.S. (1993). Integrin structure and ligant specificity in cell matrix interactions. In Molecular and Cellular Aspects of Basement Membranes, Rohrbach D.H., and Timpl R., Ed. (Academic Press, San Diego, CA), pp. 239–265.

Kupiec-Weglinski J.W., and de Sousa M. (1991). Lymphocyte traffic is modified *in vivo* by anti-laminin antibody. Immunology 72:312–317.

Lafaille J.J., Van de Keere F., Hsu A.L., Baron J.L., Has W., Raine C.S., and Tonegawa S. (1997). Myelin basic protein-specific T helper 2 (Th2) cells cause experimental autoimmune encephalomyelitis in immunodeficient hosts rather than protect them from disease. J Exp Med 186:307–312.

Lagrota-Candido J.M., Vanderlei Jr. F.H., Villa-Verde D.M.S., and Savino W. (1996). Exracellular matrix components of the mouse thymic microenvironment. V. Effects of interferon-$\gamma$ upon thymocyte/thymic epithelial cell interactions mediated by extracellular matrix ligands and receptors. Cell. Immunol. 170: 235–244.

Lane J.E., Olivares-Villagomez D., Vnencak-Jones C.L., McCurley T.L., and Carter C.E. (1997). Detection of Trypanosoma cruzi with the polymerase chain reaction and in situ hybridization in infected murine cardiac tissue. Am J Trop Med Hyg 56(6):588–95.

Lantz M., Thysell H., Nilsson E., and Olsson I. (1991). On the binding of tumor necrosis factor (TNF) to heparin and the release *in vivo* of the TNF-binding protein I by heparin. J Clin Invest 88: 2026–2031.

Lortat-Jakob H., Kleinman H.K., and Grimaud J.A. (1991). High affinity binding of interferon-$\gamma$ to a basement membrane complex (matrigel). Journal of Clinical Investigation 87:878–883.

Li Y.Q., Kobayashi M., Yuan L., Wang J., Matsushita K., Hamada J.I., Kimura K., Yagita H., Okumura K., and Hosokawa M. (1998) Protein kinase C mediates the signal for interferon-gamma mRNA expression in cytotoxic T cells after their adhesion to laminin. Immunology 93(4):455–461.

Milei J., Sanchez J., Storino R., Yu Z.X., Denduchis B., and Ferrans V.J. (1993). Antibodies to laminin and immunohistochemical localization of laminin in chronic chagasic cardiomyopathy: a review. Mol Cell Biochem 19:161–170.

Miyamoto S., Teramoto H., Coso O.A., Gutkind J.S., Burbelo P.D., Akiyama S.K., and Yamada K.M. (1995). Integrin function: molecular hierarchies of cytoskeletal and signaling molecules. J Cell Biol 131(3):791–805.

Reis D.D., Jones E.M., Tostes S., Lopes E.R., Gazzinelli G., Colley D.G., and McCurley T.L. (1993). Characterization of inflammatory infiltrate in chronic chagasic myocardial lesions: presence of tumor necrosis factor + cells and dominance of granzime A+, CD8+ lymphocytes. Am J Trop Med Hyg 48:637–644.

Ribeiro dos Santos R., Laus J.L., Silva J.S., Rossi M., Savino W., and Mengel J.O. (1992). Anti-CD4 abrogates rejection and reestablishes long-term tolerance to syngeneic newborn hearts grafted in mice chronically infected with *Trypanosoma cruzi*. J Exp Med 175: 29–39.

Romagnani S. (1996). Th1 and Th2 in human diseases. Clin Immunol Immunopathol 80: 225–235.

Sanchez J.A., Milei J., Yu Z.X., Storino R., Wenthold R. Jr., and Ferrans V.J. (1993). Immunohistochemical localization of laminin in the hearts of patients with chronic chagasic cardiomyopathy: relationship to thickening of basement membranes. Am Heart J 126:1392–1401.

Silva J.S., Morrissey P.J., Grabstein K.H., Mohler K.M., Anderson D., and Reed S.G. (1992). Interleukin-10 and interferon gamma regulation of experimental *T. cruzi* infection. J Exp Med 175:169–174.

Silva J.S., Vespa G.N., Cardoso M.A., Aliberti J.C., and Cunha F.Q. (1995). Tumor necrosis factor alpha mediates resistance to *Trypanosoma cruzi* infection in mice by inducing nitric oxide production in infected gamma interferon-activated macrophages. Infect Immun 63:4862–4867.

Silva-Barbosa S.D., Cotta-de Almeida V., Riederer I., De Meis J., Dardenne M., Bonomo A., and Savino W. (1997). Involvement of laminin and its receptor in abrogation of heart graft rejection by autoreactive T cells from *Trypanosoma cruzi*-infected mice. J Immunol 159:997–1003.

Tanaka Y., Adams D.H., and Shaw S. (1993). Proteoglycans on endothelial cells present adhesion-inducing cytokines to leukocytes. Immunol Today 14:111–115.

Timpl R., and Brown J.C. (1994). The laminins. *Matrix Biol* 14(4):275–81.

Trembleau S., Penna G., Bosi E., Mortara A., Gately M.K., and Adorin L. (1995). Interleukin 12 administration induces T

helper type 1 cells, accelerates autoimmune diabetes in NOD mice. J Exp Med 181:817–821.

Zhang L., and Tarleton R.L. (1996). Characterization of cytokine production in murine Trypanosoma cruzi infection by in situ immunocytochemistry: lack of association between susceptibility and type 2 cytokine production. Eur J Immunol 26:102–109.

Yamaguchi Y., Mann D.M., and Ruoslahti E. (1990). Negative regulation of transforming growth factor-β by the proteoglycan decorin. Nature 346:281–284.

